# High-frequency audiometry in normal hearing military firemen exposed to noise

**DOI:** 10.1590/S1808-86942010000600003

**Published:** 2015-10-19

**Authors:** Rita Leniza Oliveira da Rocha, Ciríaco Cristóvão Tavares Atherino, Silvana Maria Monte Coelho Frota

**Affiliations:** 1 MSc in Speech and Hearing Therapy - Universidade Veiga de Almeida, Head of the Speech and Hearing Therapy Clinic - Hospital Central Aristarcho Pessoa, CBMERJ. Professor and Coordinator of the Graduate Program in Speech and Hearing Therapy - Centro Universitário Celso Lisboa; Professor and Coordinator of the Graduate Program in Audiology - Universidade Veiga de Almeida; 2 PhD in Otorhinolaryngology - PUC/SP, Head of the Otorhinolaryngology Program - Hospital de Ipanema; Adjunct Professor - Rio de Janeiro State University and Universidade Veiga de Almeida; 3 PhD in Human Communications Disorders - UNIFESP, Adjunct Professor of Speech and Hearing Therapy - Federal University of Rio de Janeiro and Adjunct Professor - MSc Program at Universidade Veiga de Almeida

**Keywords:** audiology, hearing, military activities, hearing loss, noise

## Abstract

The study of high frequencies has proven its importance for detecting inner ear damage. In some cases, conventional frequencies are not sensitive enough to pick up early changes to the inner ear.

**Aim:**

To analyze the results of threshold high frequency analysis of individuals exposed to noise with normal conventional audiometry.

**Materials and Methods:**

This was a retrospective cross-sectional cohort study, in which we studied 47 firefighters of the Fire Department of Rio de Janeiro, based on Santos Dumont airport and 33 military men without noise exposure. They were broken down into two age groups: 30-39years and 40-49years. The high frequencies were studied immediately after conventional audiometry.

**Results:**

The results were most significant in the 40 to 49 years of age range, where the experimental group showed significantly higher threshold values than the control group 14000Hz (p = 0.008) and 16,000Hz (p = 0.0001).

**Conclusions:**

We concluded that noise interfered with high frequency thresholds, where all the mean values found in the experimental group were higher than those in the control group. We suggest that these data reinforce the importance of studying high frequencies, even with normal conventional audiometry in the early detection of noise-induced hearing loss.

## INTRODUCTION

According to the *National Institute for Occupational Safety and Health* (NIOSH)^1^, hearing loss induced by high sound pressure levels (HLIHSPL) is the most common irreversible occupational disease in the world. Today, there is only one legally accepted method to diagnose HLIHSPL: conventional subjective audiological evaluation. This type of hearing loss is characterized by irreversibility and the gradual progression with risk exposure time. Initially, individuals have hearing loss in one or more frequencies in the range of 3,000 and 6,000 Hz.

Frequencies above 8,000Hz are classified as high frequencies. The higher frequencies are the first to be affected in some ear diseases (presbycusis, drug-induced ototoxic effect, otitis media sequela, noise-induced hearing loss, and others (Dieroff et al.^2^; Dreschler et al.^3^, Mattews et al.^4^, Fernandes et al.^5^, Ferreira et al.^6^). One of the main clinical applications of high frequency audiometry would be the early detection of these disorders even before it shows up in conventional audiometry, when it can be considered a hearing loss.

The study of hearing loss in military personnel is nothing new, having seen that these workers are exposed to unhealthy and adverse situations. Military firefighters, for example, live through risky situations day-in-day-out; constantly exposing their lives to danger. In the state of Rio de Janeiro, besides their innumerous functions known to the population, the firemen are also responsible for the safety of airport runways throughout the state and for sea rescue in the Guanabara Bay. This population sample suffers from noise and its consequences.

The introduction of high frequency audiometry (HFA) to health inspection protocols for noise-exposed individuals could detect those workers predisposed to HLIHSPL even before they are diagnosed with changes through conventional audiometric exams, since many studies point to the increase in high frequency thresholds in workers exposed to noise^5^, ^7^, ^8^, ^9^.

Contrary to conventional audiometry, where we can classify the loss into mild to profound, high-frequency audiometry does not have standardized results. In an attempt to standardize them, Pedalini et al.^10^ studied 158 individuals with ages between 4 and 60 years without hearing complaints. The frequencies studied were 10, 12.5, 14 and 16KHz. The mean values of the tonal thresholds of these frequencies were around 10 dB HL for individuals below 30 years of age, with a gradual worsening in thresholds with aging.

In 2001, Fárfan et al.^11^ analyzed the high frequency thresholds (8 to 18KHz) from 100 volunteers between 15 and 49 years of age who did not have ear complaints and who had normal audiometric thresholds (<25 dB HL). The values found for normal thresholds were 25dB HL for the frequencies between 8 and 17KHz and 30 dB HL to 18KHz in the Amplaid 460 audiometer. The authors did not find significant differences between the right and left ears.

**Chart 1:**
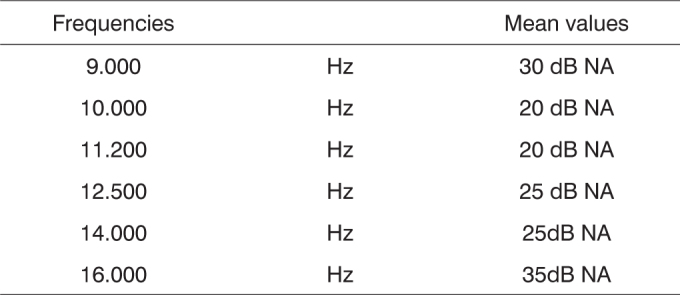
Mean value of the high-frequency audiometric thresholds of the two ears.

Although there is equipment which enables the study of high frequencies, there is a huge variability in the findings stemming from the methodology employed, equipment limitation, age range, environment characteristics, and intra-individual variability. Thus, Sahyeb et al.^12^ carried out a study with 50 individuals (24 men and 26 women) assessing these variables. We carried out four HFA assessments. Two examiners were responsible for the execution of two tests in the same day, in order to analyze intra-individual variability. On the following day, two more exams were held. Thus, there was an attempt to control the variables pertaining to daily activities of individuals. The results did not show differences between hearing sensitiveness of men and women concerning high frequency sounds, as well as between right and left ears. The results found are depicted on Chart 1.

**Chart 2:**
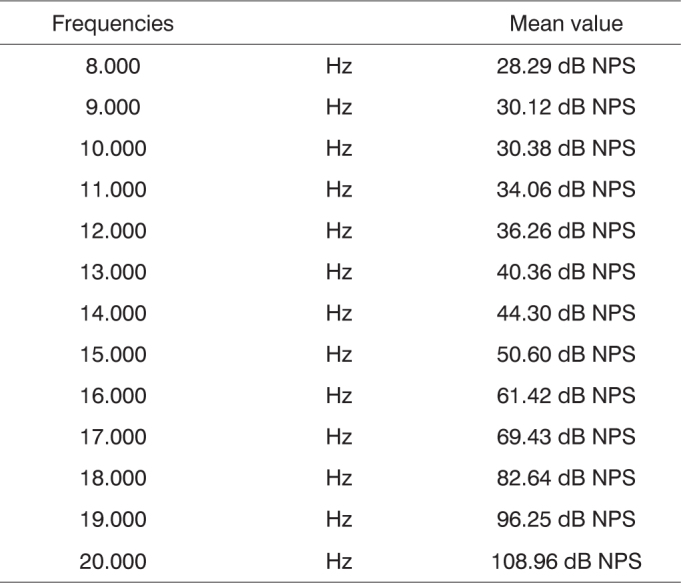
High-frequency thresholds suggested as a normality reference pattern in individuals between 30 - 40 years.

Still in search of uniformity, Martinho et al.^13^ carried out a study with 60 individuals, with the goal of tracing the high-frequency audiological profile of individuals between 30 and 40 years, with normal hearing aiming at establishing reference standards and also to establish the lower and upper limits for each frequency from both genders. The audiometer used was the Interacoustics AC-40 and HV/PRO phones. The authors noticed a drop in hearing according to increase in frequencies (Chart 2).

Recently, Sá et al.^14^ also suggested normal values for the high frequencies among individuals between 18 and 29 years. They used the *Amplaid* 460 audiometer in order to analyze 51 individuals. They did not find significant differences between males and females.

The differences in thresholds with aging do not seem so clear in conventional tonal audiometry between younger patients. Studies such as those from Baraldi et al.^15^ on hearing loss progress with aging, based on conventional audiometry, found results with greater loss in the 80-89 years of age. The authors studied individuals older than 59 years of age, whom 32.2% had normal audiometry and 28% had mild losses. In this case, conventional audiometry would not be such an efficient evaluation method as are the high frequencies for younger patients.

Although the literature has been showing differences between young and elderly individuals as to high frequency thresholds, these differences are not equally found in relation to gender and ear. Carvallo et al.^16^ conducted a HFA study in 74 individuals with ages between 18 and 30 years, 26 men and 48 women, without middle ear changes and audiometric thresholds up to 25dB between 250 and 8,000Hz. The goal of the study was to find differences between genders and ears. Among the results we did not find significant differences between males and females, although there has been a trend towards better thresholds for females. In relation to ears, in this group of individuals we did not find differences in tonal thresholds obtained from the left and right ears.

The high frequencies have also been studied in secretory otitis media^6^ and in patients complaining of tinnitus^17^. In both the studies, the high frequency thresholds can represent a diagnostic alternative on the early identification of hearing loss.

Thus, this study aims at comparing the thresholds for detecting high frequency pure tones in two groups of Military Firefighters with normal conventional audiometric values (thresholds below 25dBHL); one group of individuals without exposure to noise and another group exposed to it. The hypothesis that changes to the thresholds of high frequencies can early detect irreversible disorders of the inner ear, motivated the study of a population exposed to noise. If the tonal threshold audiometry findings of the noise-exposed group show significant differences in the high frequency thresholds in relation to the control group, this paper will reinforce the idea that the study of high frequencies can be a test used to prevent hearing loss. The reproducibility of the papers showing the importance of high frequencies in the early detection of HLIHSPL is one of the ways to insert this valuable test in occupational medicine protocols.

## MATERIALS AND METHODS

This study was a cross-sectional cohort in which we initially assessed 92 military firefighters from the State of Rio de Janeiro, assigned to the runway of Santos Dumont airport, under intense noise exposure, according to the following inclusion criteria:

- Age between 30 and 49 years;
- Male gender;
- Normal Tonal Threshold Audiometry results after 14 hours of hearing rest (thresholds below 25 dB HL from 250 to 8,000Hz);

The control group was made up of 76 firefighters with different functions in the Corps (management, healthcare, direction, filing, service and others), without exposure to intense noise, selected according to the same inclusion criteria as those in the experimental group.

Three auditory procedures caused the exclusion of 45 firefighters from the initial sample and 43 from the control group. They were taken off the study because of changes seen upon otoscopy, in the screening questionnaire and/or changes in tonal audiometry. After that, the 47 individuals in the experimental group and the 33 from the control group were submitted to high-frequency audiometry on the same day. The study group was then broken down into two subgroups according to age, namely: Subgroup 1, 23 firefighters at the age range between 30 and 39 years; Subgroup 2, ^24^ firefighters with ages between 40 and 49 years. By the same token, the control groups (33 firefighters) were: Subgroup 3, 19 firefighters with ages between 30 and 39 years; Subgroup 4, 14 firefighters with ages between 40 and 49 years.

All the procedures were done in the Central Hospital, which serves the Firefighters and their families, with the consent from the General Health Coordination of the Military Firefighters Corps of Rio de Janeiro (CBMERJ) and the Technical Supervisor of the Hospital.

Both the control and experimental groups were volunteers and were duly informed about the goals of the study, they all agreed and signed an Informed Consent Form, which was approved by the Ethics Committee after analyzing the Research Project (Resolution 88/07).

Otoscopy was carried out by the ENT Clinic; when normal, the individual was referred to the Speech and Hearing Therapy. Those individuals who had changes (ear wax, tympanic perforation, and others) were seen by the ENT consultant and taken off the study.

Afterwards, the individual would take 15 minutes to answer a questionnaire prepared by the speech and hearing therapy clinic.

The main goal of the questionnaire was to rule out any risk factor associated with hearing loss which was not occupational noise. Thus, through the questionnaire it was possible to survey the general health and auditory complaints of other firefighters and performing other functions within the corps, other past exposures, use of PPE (personal protection equipment), and complaints directly associated with the work environment.

Tonal audiometry was done after auditory rest of at least 14 hours. In vocal audiometry we employed the Speech Reception Threshold (SRT), and the Speech Recognition Percentage Index (SRPI). No individual presented vocal audiometer results different those from tonal audiometry; therefore, in such criterion nobody was excluded.

Immediately after vocal audiometry, the high frequencies were tested. The following frequencies were studied: 9,000; 10,000; 11,200; 12,500; 14,000 and 16,000KHz, where all the thresholds were retested (all the thresholds were obtained in two sequences). The frequency presentation order, choice of ear and retesting order were done randomly for each individual, so that tiredness and learning would not impact the study results.

The equipment used for tonal and vocal audiometry, as well as the one used for high frequency audiometry was the AC-40 audiometer from Interacoustics with the TDH-39P phone for conventional audiometry and the Koss HV/PRO phone for high-frequency audiometry calibrated following the ANSI S3,6 standard. The *warble* tone was chosen for the study both in conventional as well as in high frequency audiometry, because according to the data from Hamill and Haas^18^, who studied the relations between the continuous, pulsatile and warble tones in establishing the 10 and 16kHz thresholds; *warble* was the one which yielded the best thresholds in the frequencies of 14 and 16 kHz, and there were no significant differences in the remaining frequencies.

The high frequencies still do not have standardized normal results, such as tonal audiometry, for instance. For this reason, it is necessary to standardize thresholds from audiologically normal individuals (control group) with the same equipment used in the experimental group. After obtaining the results from the high frequency thresholds from the control group and from the experimental group we could compare the groups according to age: Subgroup 1 x Subgroup 3 and the Subgroup 2 x Subgroup 4. The statistical analysis comparing the thresholds between the two groups (experimental and control) was carried out by means of the *Mann-Whitney* test.

In order to check if there was a significant variation in right ear thresholds when compared to those from the left ear, we employed the Wilcoxon signaled posts test.

Non-parametric tests were used, because the thresholds did not show normal distribution (Bell-shaped distribution) because of the great data spread and/or the lack of distribution symmetry. The significance criterion used was the 5% level.

## RESULTS

In order to check whether or not there was a significant variation between the thresholds from the right to the left ear, Table 1 depicts the descriptive value (*p value*) of the statistical test (*Wilcoxon*) per group (experimental and control) and age range (30 to 39 years and 40 to 49 years), respectively. The variation between the right and left ears was calculated by the following formula: **Delta 250Hz** = (250Hz of the left year - 250Hz of the right ear).

**Table 1:** Descriptive delta level between the ears per group and age range.

	Experimental		Controle	
Frequência	30-39 anos	40-49 anos	30-39 anos	40-49 anos
Delta 9000Hz	0,53	0,15	1	0,97
Delta 10000 Hz	0,10	0,77	0,42	0,078
Delta 11200Hz	0,12	0,26	0,66	0,55
Delta 12500Hz	0,98	0,81	0,28	0,10
Delta 14000 Hz	0,44	0,77	0,60	0,55
Delta 16000 Hz	0,73	0,35	0,54	0,45

According to Table 1, there is no significant variation in the thresholds between the right and left ears by group and age range at the level of 5%.

The statistical data used to check whether or not there were significant differences in the thresholds of all the ears (right+left) between the two groups (experimental and control), stratified by age range, we used the *Mann-Whitney* test. Tables 2 and 3 provide the mean, standard deviation (SD), median, minimum and maximum thresholds of each frequency according to the group (experimental or control), and the corresponding descriptive level of the statistical test (p value) per age range (30 to 39 years and 40 to 49 years), respectively.

**Table 2:** Statistical analysis of the thresholds of all the ears according to subgroups 1 and 3 in the range of 30 to 39 years.

Frequency	group	n	Mean	S.D.	Median	Minimum	Maximum	p value
9000 Hz	experim.	48	14,1	11,9	10	0	45	0,38
	Control	38	10,0	5,3	10	0	25	
10000Hz	experim.	48	17,6	16,3	10	-5	65	0,079
	Control	38	10,5	5,4	10	0	25	
11200Hz	experim.	48	18,0	20,0	10	0	90	0,34
	Control	38	11,1	7,2	12,5	0	30	
12500Hz	experim.	48	24,8	21,8	15	0	90	0,23
	Control	38	15,9	7,2	15	5	30	
14000Hz	experim.	48	32,0	22,8	25	5	85	0,16
	control	38	22,1	8,7	20	10	35	
16000Hz	experim.	48	40,9	20,9	40	10	85	0,0009

SD: Standard Deviation

**Table 3:** Statistical analysis of the thresholds from all the ears according to subgroups 2 and 4 in the age range of 40 to 49 years.

Frequency	group	n	Mean	S.D	Median	Minimum	Maximum	p value
9000Hz	experim.	46	20,0	15,3	20	0	80	0,16
	Control	28	14,8	5,5	15	5	25	
10000Hz	experim.	46	22,9	13,9	20	5	75	0,49
	Control	28	18,8	7,0	20	0	25	
11200Hz	experim.	46	25,1	17,9	20	5	80	0,75
	Control	28	20,2	7,3	20	5	35	
12500Hz	experim.	46	33,7	21,0	30	5	90	0,31
	Control	28	26,4	7,9	25	10	45	
14000Hz	experim.	46	46,7	23,4	42,5	15	90	0,008
	Control	28	30,9	9,5	30	5	50	
16000Hz	experim.	46	60,3	19,0	60	20	95	0,0001
	Control	28	36,6	11,9	40	0	55	

S.D: Standard Deviation

**Graph 1:**
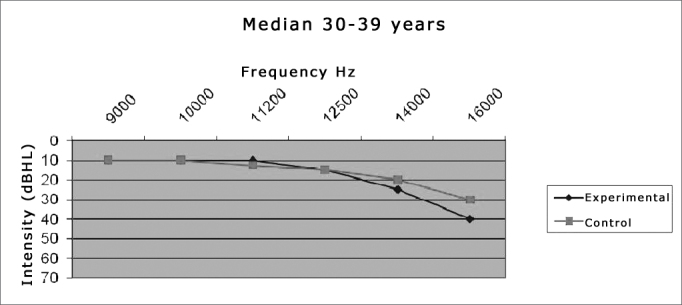
Comparison between the median value of the hearing thresholds from the experimental and control groups in the age range between 30-39 years.

**Graph 2:**
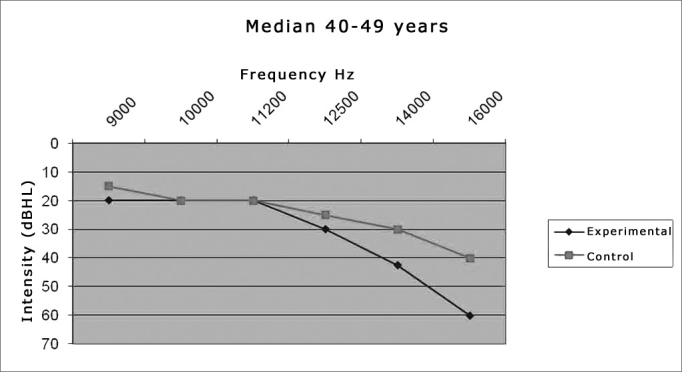
Comparison between the median value of the hearing thresholds from the experimental and control groups in the age range between 40-49 years.

Thus, the data concerning the ears could be compiled and analyzed on Tables 2 and 3 and Graphs 1 and 2.

**Graph 3:**
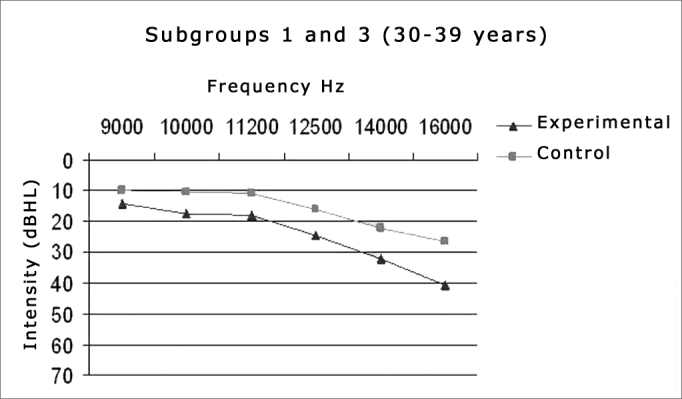
HFA mean values in the age range between 30-39 years in the control and noise-exposed groups.

**Graph 4:**
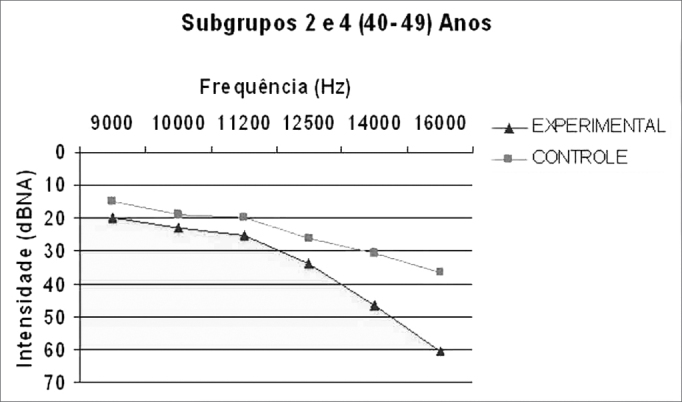
HFA mean values in the age range between 40-49 years in the control and noise-exposed groups.

We noticed a significant difference between the two groups in the frequency thresholds of the frequencies which *p* value was highlighted in **bold**, in other words, p < 0.05. We can state that:

We can notice a trend of higher thresholds in the frequency mean values of the experimental group in relation to the control group in all the frequencies concerning the two age ranges studied (Graphs 3 and 4).

The experimental group had a threshold which was significantly higher than that of the control group in the frequency of 16,000Hz (*p* = 0.0009), in the age range of 30 to 39 years (Table 2).

The experimental group had a significantly higher threshold than the control group in the frequencies of **14,000Hz** (*p* = 0.008) and **16,000Hz** (*p* = 0.0001), in the age range of 40 to 49 years (Table 3).

When we compare experimental groups and control groups, we found a worsening in the thresholds for the groups of 40-49 years in the two cases.

## DISCUSSION

There is no doubt that there are high frequency changes in auditory sensory disorders, resulting from the involvement of the cochlear basal turn. Conventional audiometry enables the investigation of pure tones in the frequencies between 250 to 8,000Hz, while high frequency audiometry helps in the investigation of the most basal cochlear responses, since it assesses hearing in the frequencies of 9,000Hz to 20,000Hz. The hearing loss found in the high frequencies could be explained by the fact that the cochlea basal region is more vascularized, which predisposes it to more evident effects of vascular damage^19^.

One of the clinical applications of high frequency audiometry is hearing monitoring in patients with or suspected of having hearing changes, such as: presbycusis, noise exposure and/or ototoxic; otitis media sequela, amongst others^2^, ^6^.

The importance of studying high frequencies has been proven along the years, by means of studies which show higher thresholds with increases in age, frequency, noise exposure, among other disorders of the middle and inner ears. Very few studies such as the one from Osterhammel et al.^20^, challenge this evidence, in which the author concluded that hearing loss in noise-exposed individuals cannot be detected through the analysis of high frequencies.

The standardization of normal high frequency thresholds is still controversial among the results found by the numerous studies which have been already carried out^12^, ^13^, ^20^, ^21^, ^22^, ^23^. Therefore, there is the need to do studies bearing the same methodology in order to better compare the data, and thus be able to get to standard thresholds. Many are the variables, such as the standardization and calibration of the equipment to be utilized (in dB SPL or HL), equipment limitations, calibration, earphone type and positioning, age range, gender differences, amongst others in order to, at the end, enable a reliable comparison between the studies.

Initially, the control group would have the same number of individuals that the experimental group; nonetheless, such number was reduced because of the difficulty in finding individuals without risks for noise-induced hearing loss. Even in health-care administrative positions, many firefighters had been previously exposed; and we took them off the control group. Because of administrative issues, those firefighters who were ruled out based on otoscopy results were not used later in the study.

Although this study did not aim at standardizing thresholds, the comparison of the control and experimental groups’ thresholds with those reported in studies carried out with these means shows values which are equal to or very close to the ones suggested by other authors^11^, ^12^, ^14^. We can stress the data from the noise-exposed group in this study, in comparison with the normality thresholds proposed by Sahyeb et al.^12^. All the thresholds from the exposed groups go beyond the values suggested for normality, even for the younger group, thus showing changes in the high frequencies in the military people from both groups of exposure. Moreover, when we compare the control group with these same values, we can say that these individuals would be within the range of values suggested as being normal for high frequencies. Therefore, the high frequency threshold values from the noise-exposed group with normal conventional audiometry results were higher than the high-frequency thresholds from the control group both in the study led by Sahyeb et al.^12^ as in the comparisons with the data present in this paper.

Results similar to those from Sahyeb et al.^12^ and the ones found in the present study were also reported by Porto et al.^9^, where the noise-exposed group obtained higher high-frequency thresholds when compared to the control group. This was the only report found with a goal similar to ours, in which they investigated two groups without a past of hearing problems showing that the high frequencies are affected even before there are drops in the conventional frequencies.

Porto et al.^9^ and Martinho et al.^13^ reported significant differences between the right and left ears’ thresholds, which was not found in our study - since according to the statistical analysis carried out by means of the *Mann-Whitney* test, significant results were not found (p<0.05) which would justify the study of the ears separately. This was also proven by studies carried out by Fernandes et al.^5^ and Carvallo et al.^16^, who also did not find differences between the high frequency tonal thresholds between the right and left ears. The study of the ears separately creates another bias concerning data standardization. The ideal thing would be to have a single standardization for both ears, following what we can already see in conventional audiometry.

The groups had to be broken down by age range because numerous papers 10, 23, 25 have already shown an increase in high frequency thresholds with aging. We would also like to report on the existence of threshold differences in the age ranges between the study groups: control x control, control x experimental and experimental x experimental. Comparing the mean values from subgroups 1 (30-39 years - experimental) and 3 (30-39 years - control) we can notice higher values in all subgroup 1 frequencies in comparison to subgroup 3. This is repeated in the following analysis, where such difference is even more striking between subgroups 2 (40-49 years - experimental) and 4 (40-49 years - control), very likely because of the more advanced age. We also found threshold increases for the older group, once again showing that the high frequencies are sensitive to aginThe findings for the high frequency values are in agreement with those from Sá et al.^14^, who also found higher thresholds for the 40-49 years-of-age group when compared to the 30-39 years individuals.

Some authors used audiometers which yielded the results in Sound Pressure Level decibels (SPL dB). Our study was carried out in Hearing Level decibels (HL dB). Thus, it was not possible to compare the values obtained from the means with most of the studies carried out by them, but we could correlate it with the shape of the resulting curves. Martinho et al.^13^, although having carried out all the studies with the same audiometer and phones we used (Interacoustics AC-40, HV/PRO phone), corrected values for SPL dB. Even then, it is possible to check the increase in mean values with the increase in frequencies for the age range between 30-40 years.

In the age range between 30 and 39 years, the results from the mean values of auditory thresholds and audiometric curve patterns showed a progressive deterioration above 9,000Hz in the experimental group, where the maximum mean value was found in the frequency of 16KHz (40.9 dB HL). This value overshadows the one suggested by Sahyeb et al.^12^, and Sá et al.^14^ concerning high frequency thresholds. Therefore, it is out of the normal standards proposed by these authors. The higher mean value obtained for the control group was 26.3 dB HL and with a median value of 30dB. These values would be in agreement with the normality values reported by prior studies^12^, ^14^ for patients with no past of noise exposure or any other risk factor for hearing loss.

The median also was the statistically significant value, where we found the one of 16,000Hz for the group between 30-39 years and those of 14,000 and 16,000Hz for the group between 40-49 years, both with the highest values for the experimental group. These findings point once again to a worsening in thresholds with increases in frequency and age, having seen that the 40-49 age group was the one which had the highest number of significant differences (p<0.05).

After the statistical analysis, other frequencies (9,000-16,000Hz) would have higher differences when the number of individuals from both groups were a bit higher (n=25 individuals) - having seen that these values would come very close to p<0.05. Moreover, we cannot neglect clinical findings, where there clearly is a trend for the thresholds to be worse for the noise-exposed group for all the frequencies above 9,000Hz.

Having seen what was stated before, we can say that high frequency audiometry can be a valuable tool for the early detection of noise-induced hearing loss, given that the thresholds of the frequencies above 8,000Hz are the first to be affected when compared to those frequencies from conventional audiometry (250-8,000Hz). These findings also demonstrate the HFA sensitivity for the early detection of noise-induced hearing loss, even in a younger group with less noise-exposure time.

## CONCLUSION

Based on the analysis of the data, we get to the end of our study with relevant data on the high-frequency threshold differences between individuals exposed or not to noise, with normal conventional tonal audiometry. We can conclude that:

- The noise exposed groups, regardless of age, showed changes in their high-frequency thresholds when compared to the control group.
- The detection of noise-induced hearing loss by HFA is more evident as age increases, but it can also be detected in younger individuals.
- With frequency and age increase there was an increase in audiometric thresholds both in conventional audiometry as well as in the high frequency one.
- No significant differences were found between the left and right ears between the frequencies of 9,000-16,000Hz.
